# P38 Re-assessment of the primary efficacy outcomes of cefepime/enmetazobactam versus piperacillin/tazobactam in adult patients with complicated urinary tract infections or acute pyelonephritis using updated EUCAST piperacillin/tazobactam susceptibility breakpoints

**DOI:** 10.1093/jacamr/dlae136.042

**Published:** 2024-08-23

**Authors:** Jon Ward, Adam Belley, Juan Quevedo, Nathalie Dunkel, Patrick Velicitat

**Affiliations:** ADVANZ Pharma, London, UK; Allecra Therapeutics, Saint-Louis, France; ADVANZ Pharma, London, UK; ADVANZ Pharma, London, UK; Allecra Therapeutics, Saint-Louis, France

## Abstract

**Background:**

In a pivotal Phase 3 clinical trial of adult patients with complicated urinary tract infections (cUTI) or acute pyelonephritis (AP), the β-lactam/β-lactamase inhibitor combination of cefepime and enmetazobactam administered by 2 h infusion demonstrated non-inferiority and met criteria for superiority compared with piperacillin/tazobactam at the primary efficacy endpoint. In this study, efficacy outcomes were reassessed when applying the updated 2021 EUCAST piperacillin/tazobactam MIC breakpoints of susceptible ≤8 mg/L and resistant ≥16 mg/L to the primary efficacy population.

**Methods:**

The primary efficacy endpoint was the proportion of patients in the microbiological modified ITT population (m-MITT) who achieved overall success (a composite of clinical cure and microbiological eradication [urine counts <10^3^ cfu/mL]) at test-of-cure. In the revised m-MITT population post-hoc analysis, patients were included who had a Gram-negative baseline pathogen with a cefepime/enmetazobactam MIC ≤8 mg/L and a piperacillin/tazobactam MIC ≤8 mg/L, which differed from the original analysis of including piperacillin/tazobactam MICs ≤64 mg/L. Two-sided 95% CI were computed using the stratified Newcombe method. The prespecified non-inferiority margin was −10% with superiority determined (when the lower bound of the 95% CI >0) in the event of confirmed non-inferiority.

**Results:**

In the revised m-MITT population, 27/345 (7.8%) patients in the cefepime/enmetazobactam group and 29/333 (8.7%) patients in the piperacillin/tazobactam group were excluded due to piperacillin/tazobactam MICs that exceeded the updated EUCAST piperacillin/tazobactam MIC breakpoints of susceptible ≤8 mg/L. Overall success occurred in 78.9% (251/318) of patients receiving cefepime/enmetazobactam compared with 60.2% (183/304) receiving piperacillin/tazobactam, with an adjusted treatment difference of 19.1% (95% CI, 11.9% to 26.1%). As clinical cure rates were similar amongst the two treatment groups, overall success in the revised cefepime/enmetazobactam group was mainly due to improved microbiological eradication.

**Conclusions:**

When applying the updated 2021 EUCAST piperacillin/tazobactam susceptibility breakpoints to primary efficacy population, cefepime/enmetazobactam was non-inferior and met criteria for superiority compared with piperacillin/tazobactam in adult patients with cUTI/AP.

